# Survey of Occupational Allergic Contact Dermatitis and Patch Test among Clothing Employees in Beijing

**DOI:** 10.1155/2017/3102358

**Published:** 2017-03-15

**Authors:** Yu-Xin Chen, Bing-Ai Gao, Hai-Yan Cheng, Lin-Feng Li

**Affiliations:** ^1^Department of Dermatology, Beijing Friendship Hospital, Capital Medical University, Beijing 100050, China; ^2^Department of Dermatology, Beijing Aerospace General Hospital, Beijing 100076, China

## Abstract

Occupational population-based epidemiological data relating to occupational contact allergies in the Chinese clothing industry are limited. To investigate the prevalence of occupational allergic contact dermatitis (OACD) and to identify the causative allergens among clothing employees in China, a cross-sectional study was conducted in 529 clothing employees at 12 clothing factories in Beijing. All employees were subjected to an interview using self-administered questionnaire and skin examination, and those who were diagnosed with occupational contact dermatitis (OCD) were patch tested. In the present survey, we found that the overall 1-year prevalence of OACD among the clothing employees was 8.5%. The 1-year prevalence of OACD among workers (10.8%) was significantly higher than that among managers (3.2%). The lesions were primarily on the hands and wrists in workers, but the face and neck in managers. The major allergens were nickel sulfate and cobalt dichloride in workers and colophony and* p*-tert-butylphenol formaldehyde resin in managers. In conclusion, workers are at a higher risk of OACD compared with managers in the Chinese clothing industry. In addition to hand dermatitis in workers, airborne contact dermatitis on the face and neck should be also addressed in managers.

## 1. Introduction

Occupational contact dermatitis (OCD) is one of the most common occupational skin diseases. However, population-based surveys on OCD in the clothing industry are limited [1, 2].

Clothing processing workers are frequently exposed to many substances, such as garment textiles, leather, adhesives, preservatives, and metal working tools (irons and scissors) [3–5]. During the processing of materials (textiles and leather) dyeing agents (such as potassium dichromate and disperse dyes), metallic elements (such as nickel, cobalt, and chrome), and finishing agents (such as colophony and epoxy resin) can affect the skin of the workers. Other processes, such as polishing, waterproofing, and sun-screening, may also involve chemicals which are considered to be potential sensitizers [6–9].

Clothing employees are exposed to the above-mentioned chemicals on garment materials and work tools, which are suspected to cause allergic contact dermatitis (CD), and may induce adverse health effects [10].

The prevalence of occupational textile dermatitis is unknown, but it appears to be increasing [3, 4]. Surveys on CD in tie and dye industry workers have been conducted, and from as early as 1985 the prevalence of CD was reported to be 16.6% [11]. In another study in 2005, Singhi et al. examined the prevalence of CD among workers engaged in the dye and textile industry around Jodhpur (Western Rajasthan), and the prevalence was reported as 7.69% [12]. Dyes are the most common cause of allergic textile CD (79.9%), whereas textile resins and formaldehyde are more often involved in occupational textile CD (13.6% and 7.6%, resp.), as found in 277 textile dermatitis patients [10].

Reports on OCD in the clothing industry have been rare over recent decades [3, 11]. However, we have found that an increasing number of workers have suspected work-related CD in clothing factories. To the best of our knowledge, few occupational population-based surveys on CD among garment employees in China have been reported to date.

The purpose of this study was to investigate the prevalence of occupational allergic CD (OACD) among employees in clothing factories in Daxing District (a suburb of Beijing) and identify the causative allergens using patch testing.

## 2. Materials and Methods

### 2.1. Study Population

Employees from 12 factories located in Daxing District, a suburb of Beijing which is the distribution center of the clothing industry in China, were recruited. The factories were selected using a quota sampling according to regional division, and Daxing District was divided into five areas, and three factories were selected from each area on the basis of convenience sampling. In all, 15 factories were contacted, and the owners of three factories refused to participate in the investigation.

The inclusion criteria were as follows: (A) factory size was between 30 and 50 employees; (B) factories were located in Daxing District in Beijing; (C) employees had worked in the garment factories for at least 1 year. Finally 529 employees in 12 factories were eligible for inclusion in the study.

Factories with between 30 and 50 employees accounted for the vast majority of the clothing factories in the survey. The employee turnover rate in the factories was high and they were a special group. In addition, employees that presented with CD in the clinic mainly came from factories with between 30 and 50 employees.

The employees were categorized into two groups: the first group consisted of sewing and ironing workers with regular and direct exposure to work materials, and the other group consisted of indirectly exposed managers, who are mainly in charge of supervision in workplace.

### 2.2. Questionnaire

A self-administered questionnaire based on the validated Nordic Occupational Skin Questionnaire (NOSQ-2002) was used [13]. The interview was conducted face-to-face in the factories and the skin of the employees was examined. The questionnaire consisted of three parts, including demographic data, details of skin complaints, and work-related conditions. Interviews were carried out to obtain information on living habits, the location and morphological aspects of the skin diseases, and the exposure to relevant substances at the workplace. Allergens with the potentially occupational exposure can derive from garment materials and work tools.

### 2.3. Skin Examination

The skin of all respondents was examined by a dermatologist with additional training on occupational contact dermatitis. The basis for a diagnosis of CD is mainly established by a comprehensive clinical history and physical examination [14].

### 2.4. Diagnosis of OCD

Evidence to support the diagnosis of OCD depends on the following well-recognized indicators: [15] (A) occupational contact with an agent known to cause similar skin changes in other individuals; (B) the occurrence of similar dermatitis in fellow workers within the same occupation; (C) a time relationship between exposure and dermatitis; (D) type and site of lesions consistent with occupational exposure; (E) similarity to other postexposure episodes of dermatitis followed by an improvement and resolution after removal. Only respondents who were verified as suffering from OCD at the examination were patch tested.

### 2.5. Diagnostic Criteria for OACD

The diagnostic criteria for OACD in the study were based on information from three sources: workplace observations, a questionnaire, and a skin examination, including the patch test results.

The diagnosis of OACD was established in cases meeting the following criteria [14–16]: (A) confirmed as a case of occupation-related CD; (B) exposure to the relevant occupational allergens; (C) confirmed positive patch test reaction to the relevant occupational allergens; and (D) exposure confirmed as a cause or as an important aggravating factor in the development of the skin diseases.

### 2.6. Patch Test

Patch tests were performed in 88 of 90 respondents with OCD using the TRUE test system (Smartpractice ApS, Hillerød, Denmark), including 74 workers and 14 managers. Consent was obtained for the 88 participants (out of the 90 participants with OCD, two managers declined to be patch tested) who were patch tested.

Those undergoing systemic or local corticosteroid or immunosuppressive treatments and those with immunocompromising disease, florid eczema, or UV exposure in the tested area were excluded.

The patch tests were applied on the back for 2 days, and readings were performed at D2 and D4 according to the guidelines of the International Contact Dermatitis Research Group. Finally, each positive reaction was interpreted, and its occupational relevance was assessed [17].

### 2.7. Ethical Issues

This study was approved by the Ethics Committee of the Friendship Hospital, Capital Medical University (number 2016-P2-029-02), and verbal consent was obtained.

### 2.8. Statistical Analysis

Statistical analyses were performed using SPSS version 20.0 (SPSS, Chicago, IL, USA). Categorical variables are presented as numbers and frequencies. Continuous variables are summarized using the median and range. The Chi-square test and Kruskal–Wallis test were used for comparison of the prevalence of CD, OCD, and OACD and positive patch test reactions to at least one of the allergens between different groups. The descriptive characteristics of the demographics and 95% confidence intervals were also used. In all analyses, *p* < 0.05 was regarded as statistically significant.

## 3. Results

### 3.1. Characteristics of the Studied Employees

**Figure 1:**
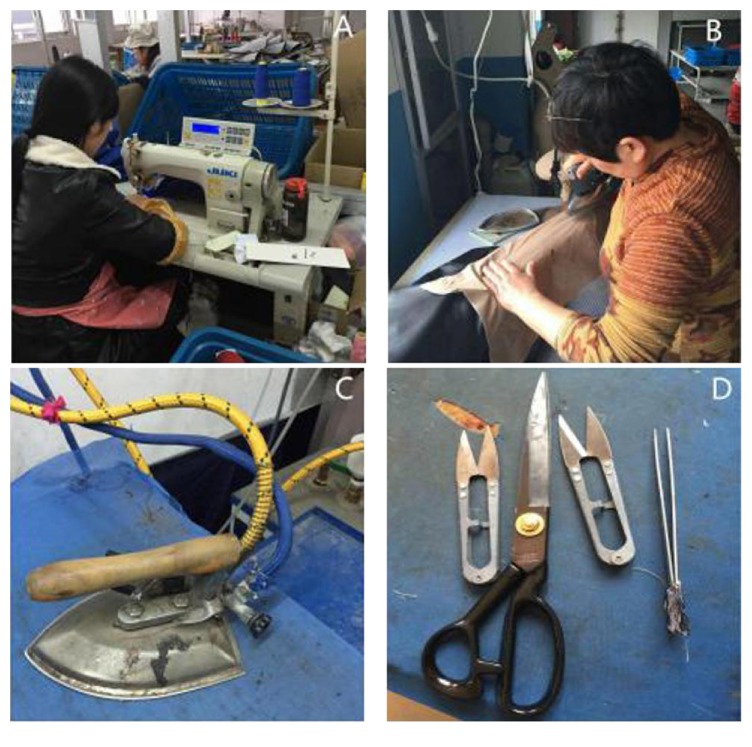
(A) Sewing worker; (B) ironing worker; (C) an iron; (D) scissors.

All 529 respondents (230 females and 299 males) employed at the 12 clothing factories were included in the study. Their mean age was 33.9 ± 8.7 years (mean ± standard deviation [M ± SD]), and the mean duration of employment in their current occupation was 10.8 ± 7.0 years (M ± SD), and their mean working hours per day were 13.2 ± 1.6 h (M ± SD). All sewing and ironing workers were directly exposed to their work materials, whereas managers were indirectly exposed to work materials (Figure 1).

Table 1 presents the demographics and occupational characteristics of the study participants. It was found that 70.3% of the subjects had direct exposure to labor materials. This group contained sewing workers and ironing workers (56.7% and 13.6%, resp.) and managers (29.7%).

### 3.2. Prevalence of OACD

**Figure 2:**
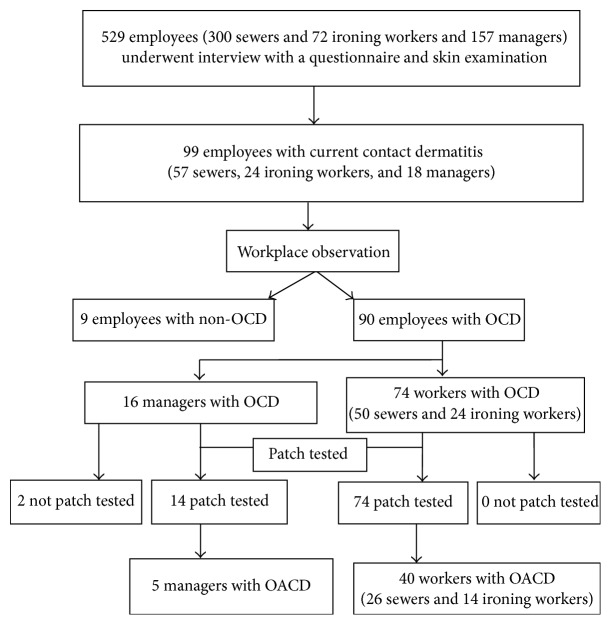
Flowchart of the 529 employees. OACD, occupational allergic contact dermatitis; OCD, occupational contact dermatitis.

Table 2 shows the prevalence of OACD, OCD, and CD in the workers and the managers. OCD was observed in 90 (17.0%) of the 529 respondents. Patch tests were performed in 88 respondents. Forty of 372 (10.8%) workers and five of 157 (3.2%) managers had a positive patch test reaction to at least one of the allergens and were diagnosed as having OACD. There was a higher prevalence of OACD in the workers than in the managers (10.8% versus 3.2%), and the difference was statistically significant (*p* = 0.004; Table 2).  Figure 2 shows a flowchart of the characteristics of the 529 respondents.

### 3.3. Location of Skin Lesions in Workers with OCD

The locations of the skin lesions in the 90 respondents with OCD are shown in Table 3. The hands, wrists, and forearms were the most frequent sites affected by OCD in the sewing and ironing workers. However, it was found that the hands were the main affected location for OCD (82.4%) in the sewing and ironing workers, but the face and neck were the most frequently affected in managers (93%), especially when airborne allergens were the causative agents.

### 3.4. Patch Test Results

A list of the standard patch test allergens of the TRUE test is shown in Table 4. It was found that the most frequent positive patch test reactions to 35 allergens were for nickel sulfate (24 workers), cobalt dichloride (21 workers), potassium dichromate (19 workers), epoxy resin (16 workers), colophony (14 workers),* p*-tert-butylphenol formaldehyde resin (13 workers), and disperse blue 106 (10 workers) in the sewing and ironing workers (*n* = 74), whereas the more common allergens for managers (*n* = 14) were colophony (4 managers) and* p*-tert-butylphenol formaldehyde resin (4 managers).

At least one positive patch test reaction to 35 allergens in the TRUE Test was observed in 88 subjects and was more frequent in sewing and ironing workers (54.1%) than in managers (35.7%), but the difference was not statistically significant (*p* = 0.208; Table 4).

More frequent positive patch test reactions are shown in Table 5. A total of 45 reactions were observed in 88 subjects and in 26 of 50 (52%) sewing workers, in 14 of 24 (58.3%) ironing workers, and in 5 of 14 (35.7%) managers.

Furthermore, the prevalence of concomitant reactions is shown in Table 6. In total, 18 (40%) of the positive patch test reactions were concomitant reactions to nickel sulfate and cobalt dichloride, but only in nine (20%) respondents were the concomitant reactions to epoxy resin and p-tert-butylphenol formaldehyde resin. The other most frequent concomitant positive reaction was colophony and p-tert-butylphenol formaldehyde resin. This was observed in 12 of 45 (28.9%) workers and in particular in four of five (80%) managers.

## 4. Discussion

Few studies have investigated the prevalence of OACD and identified causative allergens in the clothing processing industry in China. This study was conducted to evaluate the prevalence of OACD and related allergic factors among clothing workers in Beijing. From as early as 1985 [11], textile dyes and their finishing chemicals have been reported to cause CD, but only in a limited number of subjects, and several epidemic events have been reported [18, 19]. To the best of our knowledge, this study is the first epidemiological survey which targets the population of clothing workers based in China, and our results are expected to provide evidence to promote the establishment of preventative policies and an increased focus on the occupational health of clothing workers.

In the present study, the current prevalence of CD, OCD, and OACD was found to be 18.7%, 17.0%, and 5.7%, respectively. The prevalence of OACD should be higher among occupational workers. Moreover, significant differences were found in the prevalence of OCD between the lathe and ironing workers (19.9%) and the managers (10.2%; *p* = 0.007) at clothing manufacturing factories, which has not been previously reported. A speculative explanation for this may be that all workers with CD were not patch tested in previous studies [11, 12].

The available literature on the CD of workers in the clothing industry is very limited, whereby only two similar surveys on tie and dye industry workers have been previously conducted [11, 12], and in recent decades scarcely any related population-based studies on such workers have been reported. Occupationally relevant exposure in our study is similar to those in the textile dyes and leather tanning industries [20–22]. Therefore, it is possible to compare these results with those of previous studies. Mathur et al. performed a survey on the prevalence of CD among tie and dye industry workers in India in 1985, and it was found to be 16.6% (49 of 250 workers) [11]. In another study in 2005, Singhi et al. examined the prevalence of CD among workers engaged in the tie, dye, and textile industries around Jodhpur Western Rajasthan, and it was found to be 7.69% (100 of 1300 workers) [12]. Prior to this study, relevant data on the prevalence of OCD were scarce, as the literature relating the dermatitis caused by textile and leather dyes is not recent and is often not comparable, owing to differences in subject selection. In addition to different work conditions, the range of reported prevalence may be explained by the differences in the definition of OCD, the period of screening, and data collection.

In addition, the results of many cross-sectional studies on occupational diseases may be affected by a healthy-worker survivor effect. Workers who experience occupational health problems are more likely to leave high-exposure jobs, by ending employment or being transferred to another department [23].

In the current study, the hands, wrists, and forearms were the most common sites of dermatitis (68%), but a few subjects were also affected on other parts of the body, such as the face and neck in 10% of the subjects. A possible interpretation is that epoxy resin,* p*-tert-butylphenol formaldehyde resin, and colophony, regarded as volatile organic compounds, may cause airborne contact allergies at exposed sites of the body [24, 25]. In the current study, it was found that common hand dermatitis was most frequent in the sewing workers and ironing workers, but airborne contact allergy-induced face and neck dermatitis should be also monitored, especially in managers, which has not previously been reported. The trunk and the lower extremities (5%) were also affected, and a possible explanation for these atypical disease locations is inappropriate diagnoses and treatment delays [10].

In the present study, consent was obtained for 88 (98%) participants who were patch tested (out of the 90 participants with OCD, two managers declined to be patch tested), and we found that the prevalence of a contact allergy to at least one allergen was 51.1% (45 of 88 subjects) in clothing workers. However, in previously published research which derives from North America and Western Europe, in a general population, the median prevalence of contact allergies to at least one allergen was 21.2% (range 12.5–40.6%) between 1966 and 2007 [26].

Determining the occupational relevance of sensitization is essential for the diagnosis of OACD. Our study indicates that nickel sulfate (32.4%), cobalt dichloride (28.4%), potassium dichromate (25.7%), epoxy resin (21.6%), colophony (18.9%),* p*-tert-butylphenol formaldehyde resin (17.6%), and disperse blue 106 (13.5%) are more relevant as occupational sensitizers in sewing workers and ironing workers, and colophony (28.6%),* p*-tert-butylphenol formaldehyde resin (28.6%), potassium dichromate (14.3%), epoxy resin (14.3%), and formaldehyde (7.1%) were found to be more relevant in managers. The patterns of contact allergies in the workers directly exposed to work materials were different than those in managers. Studies at outpatient clinics in Germany have shown that the causative allergens in tannery workers are dichromate (3.2%), formaldehyde (1.3%), leather dyes (1.3%), and tanning agents (0.3%) [27].

Hatch reported that textile dyes were regarded as allergic contact allergens in 2003 [28]. Sensitizations were assessed in 277 textile dermatitis outpatients in Italy, and dyes (59.1%) and formaldehyde (4.5%) were the most important allergens in occupational textile CD [10].

Hatch and Maibach also reported that resins, additives, and fibers were related to textile dermatitis and reported that epoxy resin, spandex fibers, metallic fibers, wool fibers, fiber additives, formaldehyde resins, formaldehyde-releasing preservatives, and detergent residues were the causal allergens of allergic CD through the wearing or use of durable-press fabrics in 2010 [29]. In the current study, besides the above-mentioned substances, we found more important allergens, including colophony, potassium dichromate, nickel, cobalt, and chrome, which could be derived from sources such as metal tools, dyeing agents, and leather materials in the workplace. The current survey was based on a small sample population, and further studies with a larger sample size are necessary. Our results showed some differences from previous studies among different populations in different countries.

However, Hatch et al. performed patch tests using a commercial patch test series on patients suspected of textile dye dermatitis, and the results were compared with the dyes identified in that patient's submitted fabric(s). They found that dyes to which a patient was patch test-positive were infrequently identified in the fabric suspected to be the cause of the skin lesions [30]. Therefore, we should carefully interpret the clinical relevance of patch tests results. The diagnosis of OACD is mainly established using a comprehensive clinical history and physical examination, as well as patch test results [14].

In the current study, we found that sensitizations to epoxy resin,* p*-tert-butylphenol formaldehyde resin, colophony, nickel sulfate, and cobalt chloride remained concomitant, and concomitant reactions were most frequent for nickel sulfate and cobalt chloride (20.5%),* p*-tert-butylphenol formaldehyde resin and colophony (13.6%), and epoxy resin and* p*-tert-butylphenol formaldehyde resin (10.2%). Formaldehyde-based resins have been used to create permanent finishes on fabrics since the 1920s [31]. Concomitant reactions among textile dyes and finishing resins were observed in 50% of patients with textile CD in an Italian multicenter study [10]. In contrast, in the current study, we also noted formaldehyde-negative allergic reactions, whereas* p*-tert-butylphenol formaldehyde resin resulted in positive allergic reactions, in accordance with a previous report [32].

The results presented here demonstrate that around 4% of workers (21 of 529 workers) in 12 factories have allergic reactions to potassium dichromate, but over the years 1960–1969, 12% of the Swedish male workers with a chromate allergy were engaged as tannery workers [33]. We noted that some of the leather materials were processed using potassium dichromate-based dyes and found that 14.8% of subjects were sensitized to potassium dichromate. Reports on potassium dichromate sensitization are scarce in clothing manufacturing workers. However, potassium dichromate was found to be an occupationally relevant sensitizer, from a study of 472 tannery workers who were patch tested (9.2% were sensitized to potassium dichromate) [20]. Furthermore, Bregnbak et al. found that samples of leather and metal can deposit chromium on the skin at significant levels to cause contact sensitization and allergic CD [34]. Therefore, in the present study, contact allergies to potassium dichromate may possibly be derived from the leather materials and work tools (irons and scissors).

In this population, directly exposed workers and managers were sensitized to nickel sulfate and cobalt bichloride. The interpretation of nickel sulfate and cobalt bichloride patch test results remains difficult, owing to private environmental exposure [35]. The source of exposure to nickel sulfate and cobalt bichloride of the clothing factory workers in this study was probably their working tools (irons and scissors). In the present study, it was found that positive patch tests to nickel sulfate, cobalt chloride, and potassium dichromate commonly occur together. Polysensitization, defined as the number of positive reactions to a standard series of substances, for example, containing nickel, cobalt, and chromate, is also significantly associated with concurrent reactions to these metals [36], and the results of the present study are consistent with this.

In the present study, it was noted that workers who had been exposed to collodion (used with cloth) were sensitized to formaldehyde and colophony at the face and neck sites. In 1990, Lachapelle and Leroy described two cases of allergic CD to colophony, which is a component of flexible collodion [37]. Furthermore, colophony and formaldehyde are more likely to cause airborne allergic CD at exposed sites as they are volatile organic compounds, but sensitization to colophony is rarely reported in occupational populations [38, 39]. However, we found that colophony frequently induced airborne allergic CD on the face and neck of managers in clothing factories, which has not previously been reported.

## 5. Limitations and Strengths

A limitation of the present study is the use of quota sampling rather than random sampling, which may cause selection bias. Moreover, the representativeness of the study is limited to some extent because the targeted population is from small and medium-sized clothing factories in Daxing District. The strengths of the present study include a high response rate, a comparison of a group of workers with managers, and an analysis of the real working conditions among the Chinese clothing employee population. To the best of our knowledge, this is the first survey on OACD and causative allergens in the garment industry in China.

## 6. Conclusions

Clothing workers are at high risk of OACD, and this is attributed to exposure to various work materials. The patterns of contact allergies in workers are different compared with those in managers. Common hand dermatitis derived from contact allergens was more common in directly exposed workers, whereas airborne contact allergy-induced face and neck dermatitis should be addressed in indirectly exposed managers. These results are expected to provide evidence for the promotion of improvements in textile technology, the strengthening of occupational protection, and an increased focus on the occupational health of clothing workers.

## Figures and Tables

**Table 1:** Demographics and occupational characteristics of the 529 clothing employees.

	Sewing workers	Ironing workers	Managers	Total	*p* value
Number	*n* = 300	*n* = 72	*n* = 157	*n* = 529	—
Mean age (years; M ± SD)	33.3 ± 8.5	35.8 ± 7.8	34.2 ± 9.3	33.92 ± 8.7	—
Median (range)	32 (16–57)	35 (20–50)	33 (17–56)	33 (16–57)	0.062^a^
*N* (% of females)	137 (45.7%)	31 (43.1%)	63 (40.1%)	231 (43.7%)	0.523^b^
Mean duration of work (years)	10.7 ± 7.0	11.9 ± 6.5	10.5 ± 7.3	10.8 ± 7	—
Median (range)	10 (1–30)	10 (1–28)	10 (1–30)	10 (1–30)	0.204^a^
Mean working hours per day	13.9 ± 1.2	13.5 ± 1.3	11.8 ± 1.6	13.2 ± 1.6	—
Median (range)	14 (10–16)	14 (10–16)	12 (10–16)	14 (10–16)	<0.001^ac^

^a^Chi-square test.

^b^Kruskal–Wallis test.

^c^Difference between occupational groups is significant (*p* < 0.05).

**Table 2:** Prevalence (95% confidence interval) of CD, OCD, and OACD among the clothing employees.

	Sewing and ironing workers		Managers		Total		*p* value^b^
	(*n* = 372)		(*n* = 157)		(*n* = 529)		
CD^c^	21.8	0.18–0.26	11.5	0.07–0.17	18.7	0.16–0.22	0.005^a^
OCD^d^	19.9	0.16–0.24	10.2	0.06–0.16	17	0.14–0.20	0.007^a^
OACD^e^	10.8	0.08–0.14	3.2	0.01–0.07	8.5	0.06–0.11	0.004^a^

^a^Difference between occupational groups is significant (*p* < 0.05).

^b^Chi-square test.

^c^CD, contact dermatitis.

^d^OCD, occupational contact dermatitis.

^e^OACD, occupational allergic contact dermatitis.

**Table 3:** Locations of the skin complaints in the 90 clothing employees with OCD.

Locations of the lesions	Number of employees^*а*^			
	Sewing workers	Ironing workers	Managers	Total
	(*n* = 50)	(*n* = 24)	(*n* = 16)	(*n* = 90)
Hands/wrists	39	22	3	64
Forearms	19	5	12	36
Face/neck	12	1	15	29
Trunk	1	0	5	6

^*а*^More than one area can be involved in a respondent.

**Table 4:** Prevalence of contact allergies (defined as at least one positive reaction in the patch test) to 35 allergens in the TRUE Test in different groups and in total.

Substance (concentration)	Sewing and ironing workers with OCD (*n* = 74)			Managers with OCD (*n* = 14)			Total (*n* = 88)		
	*n*	Prevalence (%)	(95% CI)	*n*	Prevalence (%)	(95% CI)	*n*	Prevalence (%)	(95% CI)
Epoxy resin (0.05 mg cm^−2^)	16	21.6	0.1–0.3	2	14.3	0.04–0.4	18	20.5	0.1–0.3
Potassium dichromate (0.023 mg cm^−2^)	19	25.7	0.2–0.4	2	14.3	0.04–0.4	21	23.9	0.2–0.3
*p*-tert-Butylphenol formaldehyde resin (0.045 mg cm^−2^)	13	17.6	0.1–0.3	4	28.6	0.1–0.5	17	19.3	0.1–0.3
Nickel sulfate (0.20 mg cm^−2^)	24	32.4	0.2–0.4	1	7.1	0.01–0.3	25	28.4	0.2–0.4
Disperse blue 106 (0.05 mg cm^−2^)	10	13.5	0.08–0.23	0	0		10	11.4	0.06–0.20
Cobalt dichloride (0.02 mg cm^−2^)	21	28.4	0.19–0.39	0	0		21	23.9	0.2–0.3
*p*-Phenylenediamine (0.08 mg cm^−2^)	5	6.8	0.03–0.15	1	7.1	0.01–0.3	6	6.8	0.03–0.14
Colophony (1.20 mg cm^−2^)	14	18.9	0.1–0.3	4	28.6	0.1–0.5	18	20.4	0.1–0.3
Black rubber mix (0.075 mg cm^−2^)	3	4.1	0.01–0.11	0	0		3	3.4	0.01–0.09
Formaldehyde (0.18 mg cm^−2^)	3	4.1	0.01–0.11	1	7.1	0.01–0.3	4	4.6	0.02–0.11
Fragrance mix (0.43 mg cm^−2^)	0	0.0		0	0		0	0	
Methyldibromo glutaronitrile (0.005 mg cm^−2^)	0	0.0		0	0		0	0	
CI^+^ Me^−^ Isothiazolinone (0.004 mg cm^−2^)	0	0.0		0	0		0	0	
Imidazolidinyl urea (0.60 mg cm^−2^)	0	0.0		0	0		0	0	
Mercaptobenzothiazole (0.075 mg cm^−2^)	0	0.0		0	0		0	0	
2–Bromo–2–nitropropane–1,3–diol (0.25 mg cm^−2^)	0	0.0		0	0		0	0	
Ethylenediamine dihydrochloride (0.05 mg cm^−2^)	0	0.0		0	0		0	0	
Carba mix (0.25 mg cm^−2^)	0	0.0		0	0		0	0	
Caine mix (0.63 mg cm^−2^)	0	0.0		0	0		0	0	
Quaternium-15 (0.10 mg cm^−2^)	0	0.0		0	0		0	0	
Neomycin sulfate (0.23 mg cm^−2^)	0	0.0		0	0		0	0	
Mercapto mix (0.075 mg cm^−2^)	0	0.0		0	0		0	0	
Thimerosal (0.007 mg cm^−2^)	0	0.0		0	0		0	0	
Thiuram mix (0.025 mg cm^−2^)	0	0.0		0	0		0	0	
Diazolidinyl urea (0.55 mg cm^−2^)	0	0.0		0	0		0	0	
Quinoline mix (0.19 mg cm^−2^)	0	0.0		0	0		0	0	
Tixocortol-21-pivalate (0.003 mg cm^−2^)	0	0.0		0	0		0	0	
Gold sodium thiosulfate (0.075 mg cm^−2^)	0	0.0		0	0		0	0	
Paraben mix (1.00 mg cm^−2^)	0	0.0		0	0		0	0	
Budesonide (0.001 mg cm^−2^)	0	0.0		0	0		0	0	
Hydrocortisone-17-butyrate (0.02 mg cm^−2^)	0	0.0		0	0		0	0	
Wool alcohols (1.00 mg cm^−2^)	0	0.0		0	0		0	0	
Bacitracin (0.60 mg cm^−2^)	0	0.0		0	0		0	0	
Parthenolide (0.003 mg cm^−2^)	0	0.0		0	0		0	0	
Balsam of Peru (0.80 mg cm^−2^)	0	0.0		0	0		0	0	
At least one reaction to one allergen of TURE Test	40	54.1	0.4–0.6	5	35.7	0.2–0.6	45	51.1	0.4–0.6

**Table 5:** Prevalence of causative allergens with positive patch test reactions in the clothing employees with OACD by types of work.

Causative allergen (concentration)	Sewing workers (*n* = 50)		Ironing workers (*n* = 24)		Managers (*n* = 14)	
	No.	(%)	No.	(%)	No.	(%)
Potassium dichromate (0.023 mg cm^−2^)	16	32	3	12.5	2	14.3
Epoxy resin (0.05 mg cm^−2^)	12	24	4	16.7	2	14.3
*p*-tert-Butylphenol formaldehyde resin (0.045 mg cm^−2^)	12	24	1	4.2	4	28.6
Colophony (1.20 mg cm^−2^)	12	24	2	8.3	4	28.6
Nickel sulfate (0.20 mg cm^−2^)	10	20	14	58.3	1	7.1
Disperse blue 106 (0.05 mg cm^−2^)	9	18	1	4.2	0	0
Cobalt dichloride (0.02 mg cm^−2^)	8	16	13	54.2	0	0
*p*-Phenylenediamine (0.08 mg cm^−2^)	3	6	2	8.3	1	7.1
Formaldehyde (0.18 mg cm^−2^)	2	4	1	4.2	1	7.1
Black rubber mix (0.075 mg cm^−2^)	3	6	0	0	0	0

**Table 6:** The allergens with the most frequent concomitant positive reactions in the TRUE Test.

Concomitant reactions	Sewing workers (*n* = 50)		Ironing workers (*n* = 24)		Managers (*n* = 14)	
	Number	(%)	Number	(%)	Number	(%)
Epoxy resin (0.05 mg cm^−2^) and *p*-tert-butylphenol formaldehyde resin (0.045 mg cm^−2^)	7	14	1	4.2	1	7.1
Nickel sulfate (0.20 mg cm^−2^) and cobalt dichloride (0.02 mg cm^−2^)	5	10	13	54.2	0	0
Nickel sulfate (0.20 mg cm^−2^) and potassium dichromate (0.023 mg cm^−2^)	5	10	3	12.5	0	0
Colophony (1.20 mg cm^−2^) and *p*-tert-butylphenol formaldehyde resin (0.045 mg cm^−2^)	8	16	0	0	4	28.6
Colophony (1.20 mg cm^−2^) and epoxy resin (0.05 mg cm^−2^)	7	14	0	0	1	7.1
Potassium dichromate (0.023 mg cm^−2^) and cobalt dichloride (0.02 mg cm^−2^)	3	6	3	12.5	0	0
